# Willingness to Undergo Brain Health Testing and Motivation for Lifestyle Changes: Insights From a Survey of the Cuban Population

**DOI:** 10.1002/brb3.71358

**Published:** 2026-04-22

**Authors:** Yunier Broche‐Pérez, Zoylen Fernández‐Fleites, Diego D. Díaz‐Guerra, Marena de la C. Hernández‐Lugo, Carlos Ramos‐Galarza

**Affiliations:** ^1^ Prisma Behavioral Center Florida USA; ^2^ Psychology Department Universidad Central “Marta Abreu” De Las Villas Villa Clara Cuba; ^3^ Faculty of Psychology Pontificia Universidad Católica Del Quito Ecuador

**Keywords:** brain health, cognitive health, health motivations, lifestyle change, public health

## Abstract

**Background:**

Brain health is a critical component of overall well‐being, with early detection and lifestyle interventions playing key roles in preventing cognitive decline. While much of the research on this topic has focused on high‐income countries, less is known about public attitudes in low‐ and middle‐income settings like Cuba. This study explores the willingness to undergo brain health testing and motivation for lifestyle changes related to brain health among Cuban adults.

**Methods:**

We conducted a cross‐sectional survey with 1,049 participants aged 18 to 45 years (mean age = 24.4), using an adapted version of the Global Brain Health Survey. Participants were asked about their willingness to take a hypothetical brain health test, their intentions to change lifestyle behaviors (e.g., diet, physical activity, smoking), and factors influencing these decisions.

**Results:**

A majority of participants (73%) expressed willingness to take a brain health test, and 81.9% said they would still take the test even if it revealed an untreatable condition. While willingness to take the test was lower than in other regions, such as Europe (91%), the results highlight a proactive interest in brain health. Most participants (95.6%) were open to changing their lifestyles, particularly in improving their diet (93.4%) and increasing physical activity (95.6%). Financial constraints and limited information were identified as major barriers to lifestyle changes.

**Conclusions:**

The Cuban population shows strong interest in brain health testing and lifestyle changes to reduce cognitive risks. However, barriers such as economic challenges and limited knowledge need to be addressed. Public health campaigns should focus on providing accessible information and addressing financial constraints to promote brain‐healthy behaviors. Further research is needed to examine the long‐term impact of these attitudes on health outcomes.

## Graphical abstract

Perceived brain health risk and preventive motivation influence willingness to undergo brain health testing among adults in Cuba. These factors are also associated with intentions to engage in brain‐healthy lifestyle changes despite limited access to preventive care.

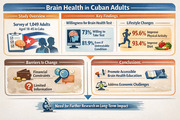

## 1 Introduction

In recent years, brain health has emerged as a critical component of overall well‐being, garnering increasing attention due to the rising global burden of brain diseases, cognitive decline, and mental health disorders (Nochaiwong et al. 2021; Pais et al. 2020; Steinmetz et al. 2024). As populations age, conditions such as Alzheimer's disease, dementia, and other neurodegenerative diseases pose significant public health challenges, not only affecting individuals' quality of life but also placing considerable strain on healthcare systems (Tay et al. 2024; Zahra et al. 2020).

While advances in medical research have provided some solutions for managing these conditions (Kumar et al. 2022; Mortada et al. 2021), prevention remains the most effective approach (Al‐Chalabi 2021; Livingston et al. 2024). Preventive strategies often focus on early detection and lifestyle interventions, including changes in diet, physical activity, social engagement, and cognitive stimulation, all of which have been shown to promote long‐term brain health (Livingston et al. 2024). Despite the growing body of evidence supporting these strategies, public engagement with brain health initiatives remains underexplored, particularly in low‐ and middle‐income countries where healthcare resources and awareness may be limited (Sexton et al. 2021; Walker and Paddick 2019).

In this context, the Cuban population represents a unique and valuable case for studying public attitudes toward brain health and the factors influencing willingness to engage in preventive behaviors. Cuba has the highest percentage of older adults in Latin America, with life expectancy exceeding 78 years. By 2030, it is expected that 30% of the Cuban population will be over 60 years old (Fernández‐Fleites et al. 2021; Llibre Rodríguez et al. 2008). However, research on the Cuban public's awareness of brain health, their willingness to undergo diagnostic tests for early detection, and their motivation to engage in lifestyle modifications aimed at preserving cognitive function remains scarce. This knowledge gap is particularly important because understanding how individuals perceive and act upon brain health recommendations is essential for developing effective health policies and interventions, particularly in populations that may not have easy access to advanced medical technologies or brain health education (Budin‐Ljøsne et al. 2022; Friedman et al. 2019; Siette et al. 2024).

In this scenario, it is crucial to explore willingness to undergo brain health testing and the motivation to adopt lifestyle changes related to brain health, especially among young and middle‐aged populations. This age group is particularly significant because the decisions and behaviors formed during youth (Farina et al. 2023) and middle age (Dohm‐Hansen et al. 2024) can have a lasting impact on brain health throughout life. Early adulthood and middle age are pivotal periods for cultivating habits and attitudes that can help reduce the risk of neurological diseases in the future.

Understanding the factors influencing this age group's willingness to take brain health tests provides valuable insights into tailoring interventions to encourage early detection and preventive behaviors. Small modifications in behavior, such as increased physical activity (Di Liegro et al. 2019), improved diet (Puri et al. 2023), reduced alcohol consumption (Daviet et al. 2022), and smoking cessation (Karama et al. 2015), have been shown to have a lasting impact on cognitive function.

In this context, the primary aim of this study was to explore the willingness to undergo brain testing and the motivation to change lifestyles related to brain health among Cuban adults. We sought to answer critical questions about individuals' attitudes and behaviors in a middle‐income country toward the prevention of cognitive decline, particularly regarding early detection and lifestyle modification.

By understanding the key motivations and barriers to adopting such behaviors, our research aimed to uncover not only the public's openness to brain testing but also the deeper psychological, social, and cultural factors that shape decision‐making regarding brain health.

This study contributes to the growing body of literature on brain health by providing a detailed examination of Cuban attitudes and behaviors related to brain health testing and lifestyle changes. By identifying factors influencing willingness to undergo brain testing and motivation to engage in health‐promoting behaviors, the findings provide valuable information for developing targeted health interventions and educational campaigns (Carver et al. 2022). Additionally, the results have broader implications for other low‐ and middle‐income countries where similar cultural and healthcare dynamics may shape public attitudes towards brain health. Ultimately, this research highlights the importance of understanding cultural and contextual factors when designing strategies to improve brain health and prevent cognitive decline, particularly in underrepresented populations.

## 2 Material and Methods

### 2.1 Study Design and Participants

This cross‐sectional study was conducted online using Google Forms. The survey link was shared through Instagram, WhatsApp, and Facebook groups. A snowball sampling approach was employed, starting with initial participants recruited via social media and community networks. These participants were then asked to refer other eligible individuals, helping to broaden the study's reach within the Cuban population aged 18 to 45.

Data collection occurred between June 30 and December 12, 2021, with 1,049 individuals voluntarily completing the survey. No financial compensation was provided to participants. The survey link was disseminated through social media platforms, including Instagram, WhatsApp, and Facebook groups, using a snowball sampling approach. Initial participants were encouraged to share the survey with other eligible individuals within their networks. Due to the open nature of this dissemination strategy, it was not possible to determine how many individuals received or viewed the survey invitation but did not participate.

### 2.2 Measures

#### 2.2.1 The Global Brain Health Survey

To explore the willingness to undergo brain health testing and the motivation to adopt lifestyle changes related to brain health, this study used the Global Brain Health Survey (Budin‐Ljøsne et al. 2020). The survey is an anonymous online questionnaire available in 14 languages, designed for individuals aged 18 and older. In this study, the authors employed a similar approach to that reported by Carver et al. (2022). Although the full GBHS consists of 16 items, the present study analyzed 9 questions relevant to decision‐making, reactions to risk information, lifestyle intentions, motivations, and perceived barriers. All items used in this manuscript are part of the original GBHS.

Below, we describe each analyzed question, including its response format and selection rules.

Willingness to undergo brain health testing was assessed by asking participants to imagine a simple brain health test to learn about their risk of developing a brain disease and to indicate whether they would wish to take such a test (**Question 1**). Responses were provided using a single‐choice, four‐point Likert scale ranging from “Yes—definitely” to “No—definitely not.” Willingness to test for unpreventable or untreatable conditions was evaluated using a similar format, asking whether participants would still take a brain health test even if it provided information about a disease that could not be prevented or treated.

Participants who expressed willingness to take a brain health test were then asked about their reasons for doing so (**Question 2**). This item allowed multiple responses and included motivations such as obtaining information about cognitive and mental health, determining the risk of developing a brain disease, responding if found to be at risk, and preparing for the future. Conversely, participants who indicated unwillingness to take a brain health test were asked to report their reasons for not doing so (**Question 3**). This multiple‐response question included options such as not wanting to worry about something that might not happen, not wanting to know about an untreatable disease, fear of the results, or the belief that nothing could be done to improve brain health (**Question 4**).

Likely reactions to receiving brain health risk information were assessed by asking participants to imagine that a brain health test revealed an increased risk of developing a brain disease and to indicate their most likely responses (**Question 5**). Participants rated several potential reactions, including seeking professional help, consulting family or friends, searching for information online or in libraries, changing their lifestyle, and planning for the future. Each reaction was rated separately using a four‐point Likert scale ranging from “Definitely yes” to “Definitely not.” For analytical purposes, responses of “Definitely yes” and “Fairly likely” were combined, in line with previous GBHS studies.

Willingness to change specific lifestyle behaviors was evaluated by asking participants how likely they would be to adopt various lifestyle changes if advised by a physician that such changes could reduce their risk of developing a brain disease (**Question 6**). Participants rated their likelihood of changing eating habits, exercising more, improving sleep, engaging in relaxing activities, participating in brain‐stimulating activities, reducing alcohol consumption, reducing smoking, and increasing socialization. Each behavior was rated independently using the same four‐point Likert scale. Analyses focused on participants who reported not already engaging frequently in each behavior.

Motivations for lifestyle change were assessed by asking participants what factors would motivate them to change their lifestyle to improve brain health (**Question 7**). This was a multiple‐response question and included options such as experiencing more frequent brain‐related problems (e.g., memory loss or difficulty concentrating), receiving a diagnosis of a brain disease, having a close friend or relative diagnosed, receiving advice from a doctor, support from family or friends, knowing that lifestyle changes are beneficial, and the availability of interventions that are easy to follow or affordable.

Perceived barriers to lifestyle change were measured by asking participants what would prevent them from changing their lifestyle for their brain health (**Question 8**). This multiple‐response item included barriers such as lack of information, lack of motivation, lack of time, financial costs, having to give up enjoyable activities, uncertainty about whether changes would be beneficial, needing to engage in activities they do not enjoy, or having to make changes alone.

In addition to the main survey items, participants answered several background questions related to brain health, including how often they think about their brain health, whether they have previously cared for a family member with a brain disease, prior participation in brain health research, and the presence of any disability or illness affecting work (**Question 9**). These items were used to characterize the sample and explore associations with willingness and reactions to brain health testing.

Standard demographic variables were also collected, including age (18–25, 26–35, and 36–45 years), gender, educational level, employment status, and healthcare experience. Appendix 1 shows the survey and answer options.

#### 2.2.2 Procedure

All participants provided informed consent before participation. The protocol received approval from the Human Subjects Institutional Review Board (HSIRB) of the Universidad Central “Marta Abreu” de Las Villas, Department of Psychology (ethics protocol number: HSIRB‐2021‐016). The research adhered to the ethical standards outlined in the 1964 Declaration of Helsinki.

#### 2.2.3 Data Analysis

The analysis began with descriptive statistics, which summarized and outlined the key characteristics of the collected data. We employed measures of central tendency, primarily the mean, to assess the average responses across survey items. This approach provided a clear overview of the dataset's general patterns and trends.

In addition, we calculated percentages to illustrate the distribution of responses to key survey questions. This helped highlight the proportion of participants who held specific views or exhibited certain behaviors, enabling a more detailed interpretation of the results. To examine factors believed to influence brain health across different demographic groups, we used binomial and multinomial logistic regression with the enter method, using the first category as the reference. Preliminary checks were performed to ensure that the assumptions of linearity, independence of errors, and multicollinearity were not violated (Field 2018). The final analysis was conducted using 1,049 valid cases, and data processing was carried out using SPSS software version 26.

The dataset, along with the adapted questionnaire for the Cuban population and a codebook, is publicly available online (Broche‐Pérez et al. 2024).

## 3 Results

### 3.1 Respondent Characteristics

A total of 1,049 participants completed the adapted Global Brain Health Survey. The mean age of the sample was 24.4 years (SD = 6.49), with ages ranging from 18 to 45 years. The predominant age group was 18 to 25 years, comprising 70.4% of participants (Table 1).

**TABLE 1:** Demographic characteristics of the sample (*n*_=_ 1049).

Variable	Fre.	%
**Age**		
18‐25	738	70.4 %
26‐35	220	21.0 %
36‐45	91	8.7 %
**Gender**		
Female	692	66.0 %
Male	357	34.0 %
**Education**		
Primary education	2	0.2 %
Middle school	11	1.0 %
High school	604	57.6 %
University degree	432	41.2 %
**Employment**		
Full‐time employment	342	32.6 %
Full‐time student	420	40.0 %
Housewife	186	17.7 %
Unemployed	43	4.1 %
Volunteer work	52	5.0 %
Disability (can't work)	6	0.6 %

*Note*. Frec.(frequency).

Regarding gender distribution, 66% of respondents identified as female. Geographically, participants were primarily from Cuba's central region, accounting for 78% of the sample. Regarding educational background, 57.6% completed high school, while 41.2% held a university degree. Employment status revealed that 32.6% of participants were employed full‐time, whereas 40% identified as full‐time students (Table 1).

When asked how often they think about their brain health (Question 9), 28.4% reported rarely or never thinking about it. Additionally, a significant majority (67.9%) indicated that they have never acted as a caregiver for someone with a brain disease, and 87.3% reported no prior participation in research related to brain health. Finally, 76.5% of the sample reported having no disabilities or illnesses, suggesting a generally healthy population in terms of physical and cognitive conditions.

### 3.2 Willingness to Undergo Brain Testing

In response to *“Imagine a simple brain health test to learn about the risk of developing a brain disease. Would you wish to take such a test?”* (Question 1) 73% of participants indicated they would be willing to take the test (responding “probably” or “definitely”), while 27% expressed that they would not wish to take it (responding “probably” or “definitely” not).

A clear majority of participants expressed willingness to undergo brain health testing even if the results indicated a disease that could not be prevented or treated (Question 2). Specifically, 57.96% of respondents indicated that they would *definitely* take such a test, while an additional 23.83% reported that they would *probably* do so. Together, this represents 81.8% of the sample expressing a positive inclination toward testing despite the absence of preventive or therapeutic options. In contrast, 12.30% of participants reported that they would *probably not* take the test, and 5.82% indicated that they would *definitely not* do so.

According to age group, people with 26 years or more have a higher probability of willingness to take a test for brain health (26‐35 years, OR = 1.70, 1.14‐2.54; 36–45 years, OR = 2.15, 1.18‐3.93) and unpreventable diseases (36‐45 years, OR = 2.53, 1.16‐5.50), in comparison with the younger group of people. People with healthcare experience have 1.5 times more probability of willingness to test for unpreventable or untreatable diseases in comparison with those without experience (Table 2).

**TABLE 2:** Willingness to take a test.

		For brain health		Unpreventable or untreatable diseases	
Variable	Characteristics	OR	99% CI	OR	99% CI
Gender	Women				
	Men	−0.77	0.57‐1.02	−0.89	0.63‐1.24
Age (years)	18‐25				
	26–35	1.70*	1.14‐2.54	1.5	0.94‐2.37
	36–45	2.15*	1.18‐3.93	2.53*	1.16‐5.50
Education	Lower education				
	Higher education	0.92	0.67‐1.27	1.03	0.71‐1.48
Cared for family with a brain disease	No				
	Yes	0.99	0.74‐1.34	0.78	0.56‐1.1
Healthcare experience	No				
	Yes	1.25	0.94‐1.66	1.5*	1.08‐2.09

**FIGURE 1:**
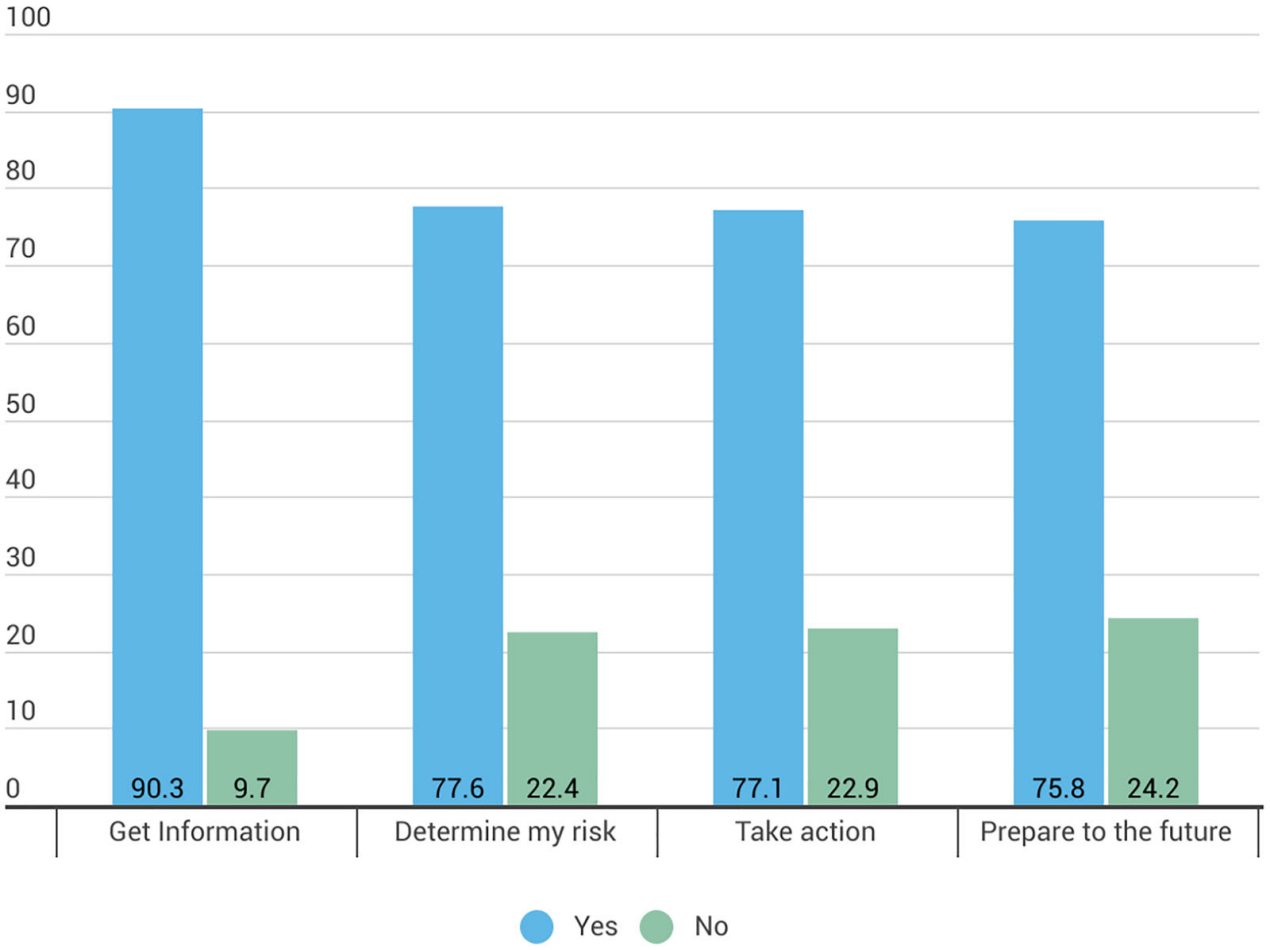
Reasons why participants would take a test to learn about the risk of developing a brain disease (the values are expressed in percent). Participants could select multiple responses.

Among the participants willing to take a brain health test (Question 3), the most common reason was to gain information about their cognitive and mental health, with 90.3% selecting this option. 77.6% of participants indicated they would take the test to determine their risk of developing a brain disease, and 77.1% expressed willingness to take action if they were at risk, such as making lifestyle changes, seeking counseling, or starting treatment. Additionally, 75.8% stated they would take the test to prepare for the future, including informing family members about potential risks (Figure 1).

Men were less likely to take a brain health test to determine the risk of developing a brain disease (OR = ‐0.72;.54‐.97) than women. People with healthcare experience were more likely to take a test to get information about cognitive and mental health (OR = 1.29, 1.01‐1.66) (Table 3).

**TABLE 3:** Reasons why participants would take a test.

		To get information about my cognitive and mental health		To determine my risk of developing a brain disease		To respond if I am at risk	
Variable	Characteristics	OR	99% CI	OR	99% CI	OR	99% CI
Gender	Women						
	Men	1.01	0.77‐1.31	−0.72*	0.54‐.97	−0.83	0.64‐1.08
Age (years)	18‐25						
	26–35	1.13	0.8‐1.58	1.04	0.72‐1.51	1.3	0.92‐1.83
	36–45	1.04	0.65‐1.66	1.18	0.72‐1.94	0.93	0.59‐1.49
Education	Lower education						
	Higher education	1.02	0.77‐1.36	‐.94	.69‐1.29	1.09	0.82‐1.45
Cared for family with a brain disease	No						
	Yes	−0.85	0.65‐1.11	1.09	0.82‐1.45	0.98	0.75‐1.27
Healthcare experience	No						
	Yes	1.29*	1.01‐1.66	1.19	0.90‐1.56	1.11	0.86‐1.42

**FIGURE 2:**
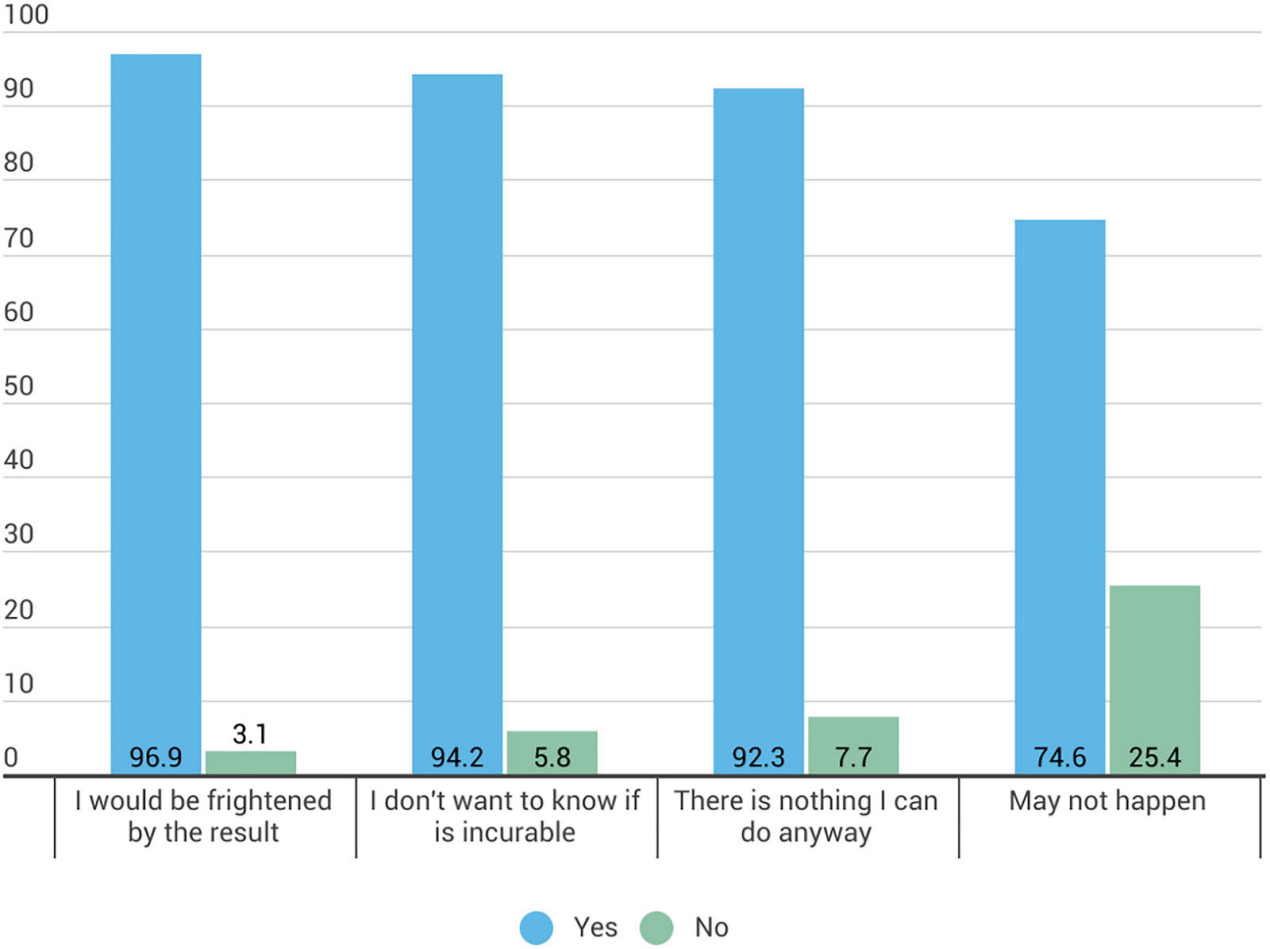
Reasons why participants would not take a test to learn about the risk of developing a brain disease (the values are expressed in percent). Participants could select multiple responses.

Among the participants unwilling to take a brain health test (Question 4), the most prevalent concern was the desire to avoid worrying about something that may not happen, with 96.9% citing this as a key reason. 94.2% of respondents expressed reluctance to know about a disease that could not be prevented or treated, while 92.3% indicated that the fear of receiving frightening or unsettling results would deter them. Lastly, 74.6% of participants felt that there was nothing they could do to improve their brain health regardless of the test results, contributing to their decision not to undergo testing (Figure 2). Men and individuals with higher educational levels were more likely to be unwilling to undergo a brain health evaluation due to not worrying about something that might not happen (OR = 1.85, 1.17‐2.92; 1.86, 1.13‐3.07), compared to women and those with lower levels of education. In contrast, individuals aged 26–35 were less likely to avoid taking a test due to fear of the results (OR = ‐0.44, 95% CI: 0.19–0.98) compared to the youngest age group (Table 4).

**TABLE 4:** Reasons why participants would not take a test.

		I do not want to worry about something that may not happen		I do not want to know about a disease that could not be prevented or treated		I would be frightened by the result		There is nothing I can do for my brain health anyway	
Variable	Characteristics	OR	99% CI	OR	99% CI	OR	99% CI	OR	99% CI
Gender	Women								
	Men	1.85**	1.17‐2.92	1.4	0.91‐2.14	−0.56	0.31‐1.01	1.81	0.72‐4.56
Age (years)	18‐25								
	26–35	−0.73	0.40‐1.33	−0.79	0.43‐1.44	−0.44*	0.19‐0.98	−0.81	0.23‐2.79
	36–45	−0.64	0.26‐1.59	−0.4	0.15‐1.27	−0.47	0.16‐1.40	0	
Education	Lower education								
	Higher education	1.86*	1.13‐3.07	−0.95	0.58‐1.54	1.26	0.72‐2.22	1.05	0.37‐2.99
Cared for family with a brain disease	No								
	Yes	1.18	0.74‐1.89	−0.92	0.58‐1.45	−0.61	0.34‐1.11	2.12	0.84‐5.34
Healthcare experience	No								
	Yes	−0.73	0.46‐1.16	−0.58	0.38‐.91	−0.83	0.50‐1.37	−0.48	0.18‐1.31

### 3.3 Motivation for Change

When posed with the question, “Imagine you undergo a brain health test and it shows that you have a risk of developing brain disease. What would be your most likely reaction?” (Question 5), participants indicated a strong likelihood of taking various actions. Specifically, 98.7% stated they would seek professional help, 89.1% would consult their family for advice, and 93.9% expressed a desire to seek more information about their condition. Additionally, 95.6% indicated they would be willing to change their lifestyle if necessary, and 91.4% stated they would plan for the future in response to the test results.

Men were less likely than woman to react by seeking information online/at the library, changing their lifestyle, or planning the future (OR = ‐0.58, 0.34‐0.98; OR = ‐0.3, 0.16‐0.61; OR = 0.61, 0.39‐0.95). People 26–35 years old were more likely than younger people to react by changing their lifestyle or planning the future (OR = 4.49, 1.29‐15.67; OR = 3.16, 1.29‐7.71). People with higher education were also more likely to react by making plans for the future (OR = 2.09, 1.16‐3.76) in comparison to those with a lower educational level (Table 5).

**TABLE 5:** Likely reactions to test results on brain health risk.

		I would seek professional help (e.g., my doctor)		I would seek advice from family and friends		I would seek information online/at the library		I would change my lifestyle if required		I would plan for the future	
Variable	Characteristics	OR	99% CI	OR	99% CI	OR	99% CI	OR	99% CI	OR	99% CI
Gender	Women										
	Men	−0.70	0.3‐1.64	−0.78	0.52‐1.17	−0.58*	0.34‐.98	−0.3***	0.16‐.56	−0.61*	0.39‐.95
Age (years)	18‐25										
	26–35	−0.76	0.26‐2.25	1.14	0.66‐1.99	2.07	0.87‐4.94	4.49*	1.29‐15‐67	3.16*	1.29‐7.71
	36–45	1.74	0.21‐14.33	1.71	0.70‐4.18	1.04	0.38‐2.84			3.51	0.82‐14.99
Education	Lower education										
	Higher education	1.15	0.43‐3.08	1.13	0.71‐1.79	1.2	0.65‐2.21	1.05	0.51‐2.15	2.09*	1.16‐3.76
Cared for a family with a brain disease	No										
	Yes	−0.73	0.31‐1.72	−0.95	0.63‐1.44	1.36	0.75‐2.44	1.17	0.59‐2.34	−0.92	0.57‐1.49
Healthcare experience	No										
	Yes	1.02	0.44‐2.39	−0.84	0.56‐1.25	1.18	0.70‐2.00	1.81	0.94‐3.52	1.36	0.86‐2.15

**FIGURE 3:**
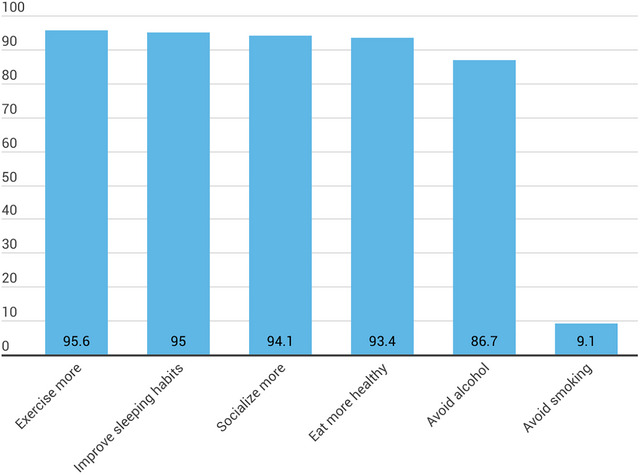
Lifestyle variables that participants would change if diagnosed with a brain disease (values expressed as percentages). Participants could select multiple responses.

In response to the question, “Your doctor tells you that you can reduce your risk of developing a brain disease by changing your lifestyle: How likely are you to do any of the following?” (Question 6), the analysis focused on participants who do not currently engage in these activities frequently (Table 4). Among these respondents, 93.4% indicated they would modify their eating habits, 95.6% expressed a willingness to exercise more, and 95% stated they would aim to sleep better. Additionally, 85% reported they would engage in more relaxing activities, while 95% indicated they would participate in more brain‐stimulating activities. Furthermore, 86.7% expressed a desire to reduce alcohol consumption, although only 9.1% indicated they would reduce smoking. Lastly, 94.1% stated they would socialize more as part of their lifestyle changes (Figure 3).

When asked, *“What would motivate you to change your lifestyle to improve brain health?”* (Question 7), participants identified several key factors. A notable 73.8% indicated that the increasing frequency of brain problems, such as memory loss, difficulty focusing, or disorientation, would motivate them to make changes. Additionally, 55.8% stated that a diagnosis of a brain disease alone would be motivating.

Regarding the nature of interventions, 15.9% emphasized the importance of treatments or lifestyle changes that are fun or easy to follow, while 19.5% emphasized the need for these changes to be affordable. The diagnosis of a close friend or relative with a brain disease would motivate 26.6% of participants, and 31.7% indicated that receiving advice from a doctor based on their risk of developing a brain disease would prompt them to take action. Support from family and friends was seen as motivating by 15.4% of respondents, while 27.7% noted that knowing lifestyle changes are beneficial would encourage them to make those changes.

When asked, *“What would prevent you from changing your lifestyle for your brain health?”* (Question 8), participants identified several barriers. The most frequently mentioned obstacle was a lack of information about what to do, with 45.6% citing this as a significant deterrent. Following closely, 43.6% indicated that a lack of motivation would hinder their efforts, while 42.3% noted that a lack of time would pose a challenge.

Financial concerns also emerged as a barrier, with 24.4% expressing that if making changes were expensive, such as requiring a gym membership, it would prevent them from acting. Additionally, 25.9% stated that having to give up activities they enjoy would deter them, and 23.5% mentioned uncertainty about whether the changes would be beneficial. Lastly, 16.3% expressed concerns about needing to take up activities they do not enjoy, and only 8.7% indicated that making changes alone would prevent them from pursuing lifestyle modifications.

## 4 Discussion

Our study aimed to explore the willingness to undergo brain health testing and the motivation to change lifestyles related to brain health within the Cuban population. The results revealed a significant openness toward brain health testing, with 73% of participants indicating they would probably or definitely take a hypothetical brain health test. Notably, even in the face of the potential for untreatable or unpreventable conditions, 81.9% of participants still expressed a willingness to undergo testing.

This suggests a strong desire for knowledge and proactive engagement in health management, echoing the findings of similar studies in other regions. For instance, Carver et al. (2022) found that over 91% of participants in Europe expressed a willingness to take a brain health test, with 86% willing to do so even if it provided information on conditions that were irreversible or untreatable. The slight difference in willingness between our sample and that of Carver et al. (2022) could be attributed to socio‐cultural factors, healthcare access, and varying levels of awareness about brain health across populations. These differences highlight the importance of tailoring public health interventions and educational campaigns based on cultural and regional contexts, ensuring that information about brain health testing resonates with diverse populations.

Our study also revealed important insights into participants’ motivation to change lifestyle behaviors in response to the risk of brain health issues. A majority of participants indicated they would seek professional help upon learning of a potential risk for brain disease, demonstrating a proactive attitude toward health management. This finding is consistent with previous research that underscores the value of fostering an environment where individuals feel empowered to engage with healthcare professionals about cognitive concerns. The strong inclination toward involving family members and seeking additional information about health conditions also reflects the importance of social support systems in health decision‐making (Reisinger et al. 2018), which has been shown to improve health outcomes (Costa‐Cordella et al. 2021; Wright 2016), particularly in contexts where communal values play a key role in daily life.

One of the most striking results was the high willingness to make lifestyle changes to improve brain health. A substantial 95.6% of participants indicated a readiness to change their lifestyle, with most expressing a desire to alter their eating habits (93.4%) and increase physical activity (95.6%). These findings align with existing research emphasizing the role of diet and exercise in promoting cognitive health and preventing neurodegenerative diseases (Pahlavani 2023; Puri et al. 2023).

These results highlight an opportunity for public health campaigns to leverage the existing recognition among individuals about the importance of lifestyle choices for cognitive function. In particular, the desire to make dietary changes suggests a potential for health interventions focused on promoting brain‐healthy foods, which could be integrated into existing public health campaigns.

Our findings revealed a marked discrepancy between participants’ reported willingness to reduce alcohol consumption (86.7%) and smoking (9.1%). This difference should be interpreted with caution. Smoking status was not directly assessed in the survey; therefore, the low endorsement of smoking reduction may reflect a relatively low prevalence of smokers within the sample rather than a lack of motivation to reduce smoking among smokers specifically. This interpretation is plausible given that the sample consisted largely of young adults and students, a group in which smoking prevalence may be lower than in older populations. Future studies should explicitly assess smoking behavior to allow for more precise interpretation of lifestyle change intentions.

Beyond sample composition, this pattern may also reflect broader societal attitudes toward alcohol and tobacco. Alcohol‐related health risks tend to be more visible and socially discussed, whereas smoking cessation continues to represent a substantial public health challenge worldwide, shaped by physical dependence as well as social and behavioral factors (Bitar et al. 2023; Coleman et al. 2024). Accordingly, public health strategies aimed at promoting brain health may benefit from more tailored approaches to smoking cessation that address both addiction‐related mechanisms and contextual influences on smoking behavior.

Furthermore, our study identified several barriers to lifestyle changes, including limited information, time constraints, and financial concerns. These findings are in line with previous research highlighting the critical role of accessible and clear information in motivating health behavior changes (Matthews et al. 2024; Michaelsen and Esch 2023). Specifically, financial barriers were a significant concern, with 24.4% of participants citing the high cost of lifestyle changes, such as gym memberships or healthy foods, as a deterrent. In Cuba, where economic constraints are a pressing issue, these barriers are even more pronounced.

The ongoing economic crisis, with high inflation and shortages of essential goods (Kunkel 2024), makes it challenging for individuals to adopt healthier lifestyles, especially when those changes are perceived as luxuries rather than necessities. Structural socioeconomic inequalities have exacerbated brain‐age gaps, especially in Latin American and Caribbean (LAC) countries, where being from the region is linked to accelerated brain aging (Moguilner et al. 2024). This pattern is also applied to the Cuban population. The ongoing economic crisis is likely to affect brain health not only in the short term but also over the coming decades, potentially leading to a rise in the incidence of brain diseases, particularly neurodegenerative disorders.

This context calls for the development of low‐cost or community‐based programs aimed at promoting brain health, particularly for individuals from lower socioeconomic backgrounds. Providing affordable, accessible options for physical activity and healthy eating would be a crucial step in ensuring that all individuals, regardless of financial status, have the opportunity to engage in brain‐healthy behaviors.

Lastly, the motivation to change lifestyle behaviors was strongly driven by personal experiences, with 73.8% of participants citing the increasing frequency of brain‐related issues like memory loss or disorientation as a key motivator. This finding underscores the importance of raising awareness about the early signs of cognitive decline, which may encourage individuals to take preventive actions before conditions become more serious (Cappa et al. 2024). Public health campaigns that focus on early detection and the role of lifestyle in brain health could be more effective if they highlight these early signs and connect them to concrete steps that individuals can take to protect their cognitive function.

### 4.1 Limitations

While this study provides valuable insights into willingness to undergo brain health testing and intentions to adopt lifestyle changes within the Cuban population, several limitations should be acknowledged. First, the cross‐sectional design precludes causal inference. Although associations were observed between attitudes toward brain health testing and reported intentions for lifestyle change, it is not possible to determine whether these attitudes translate into sustained behavioral change over time or are directly influenced by specific interventions, experiences, or external factors.

Second, the study relied on an online survey disseminated through social media platforms using a snowball sampling strategy. This recruitment approach introduces self‐selection bias, as individuals who choose to participate may differ systematically from those who did not, particularly with respect to interest in health‐related topics, digital access, or prior exposure to health information. As a result, the findings should not be interpreted as nationally representative but rather as exploratory and hypothesis‐generating insights into brain health perceptions among a large segment of Cuban adults. In addition, because the survey link was openly circulated, it was not possible to determine how many individuals received or viewed the invitation but did not participate, and therefore a response rate could not be calculated. This limitation is inherent to Google Forms–based and other open online survey methodologies.

Third, the sample consisted predominantly of young adults, with a mean age of 24.4 years, which may limit the generalizability of the findings to older adults who are more directly affected by cognitive decline and neurodegenerative conditions. Given that concerns about brain health often increase with age, future research should include a broader age range to better capture perspectives across the lifespan, particularly among middle‐aged and older adults or individuals with increased neurological risk due to family history or health status.

Another important limitation relates to the measurement instrument itself. The Global Brain Health Survey was originally developed and applied primarily in European populations and included a substantial proportion of older adults, many of whom had prior involvement in brain research. Although the survey items were conceptually relevant and understandable in the present Cuban sample, some questions may not have fully captured age‐specific, cultural, or socioeconomic factors that influence perceptions of brain health among younger adults living in low‐ and middle‐income contexts. The extent to which certain items resonated equally across age groups and cultural settings could not be formally assessed in this study. Future research would benefit from culturally and developmentally adapted versions of the instrument, as well as formal validation studies in diverse populations, to improve sensitivity to contextual differences in brain health attitudes and decision‐making.

Finally, the study relied on a single self‐report survey to assess attitudes and intentions, which may not fully reflect the complexity of participants’ motivations and behavioral decision‐making processes. Factors such as personal experiences, cultural norms, socioeconomic constraints, and contextual influences likely play an important role in shaping health‐related behaviors but were not examined in depth. Mixed‐methods or qualitative approaches could provide a more nuanced understanding of the psychological and social mechanisms underlying willingness to engage in brain health testing and preventive lifestyle changes.

### 4.2 Future Directions

Given the limitations of our study, several directions for future research in this area can be suggested. First, longitudinal studies would provide more robust evidence regarding the causal relationships between attitudes toward brain health testing and subsequent lifestyle changes. Tracking individuals over time could help clarify whether taking a brain health test leads to sustained changes in behavior, such as improvements in diet, exercise, and smoking cessation. This would also help to assess the long‐term impact of early brain health assessments on cognitive function and overall health.

Second, diversifying the sample to include a wider age range and different demographic groups would provide a more comprehensive understanding of how attitudes toward brain health vary across the lifespan. This could be especially important in countries with aging populations, where the risks of neurodegenerative diseases are more prevalent. Future studies should also examine the specific needs and attitudes of older adults or individuals at higher risk for cognitive decline, including those with a family history of neurological conditions.

Qualitative research could also complement the quantitative findings from this study by exploring in‐depth personal experiences, cultural perceptions, and the social dynamics that influence individuals' willingness to engage in brain health testing and lifestyle changes. Interviews or focus groups could provide richer insights into participants’ decision‐making and highlight potential barriers and facilitators that quantitative surveys may not fully capture.

Additionally, more research is needed to explore the economic barriers to adopting brain‐healthy behaviors, particularly in countries like Cuba, where financial constraints are a significant challenge. Investigating how individuals perceive the affordability of lifestyle changes (e.g., gym memberships, healthy food options) and the role of government‐ or community‐driven interventions could provide valuable insights into making brain health‐related behaviors more accessible to underserved populations. Public health strategies could be further developed by understanding these barriers and exploring low‐cost or culturally appropriate solutions.

## 5 Conclusion

In conclusion, while our findings indicate a strong willingness among the Cuban population to engage with brain health testing and make lifestyle changes, several challenges remain, particularly around information accessibility, cultural factors, and economic constraints. Future interventions should focus on addressing these barriers while leveraging the existing motivation for change. Furthermore, targeted health communication strategies that involve family and community networks, as well as the integration of affordable lifestyle interventions, could help enhance the impact of brain health initiatives in Cuba and similar low‐ and middle‐income settings.

## Author Contributions

**Yunier Broche‐Pérez**: conceptualization, data curation, methodology, data analysis, supervision, writing – review and editing, writing – original draft. **Zoylen Fernández‐Fleites**: conceptualization, data curation, data analysis, validation. **Diego D. Díaz‐Guerra**: conceptualization, data curation, validation, writing – review and editing, project administration, supervision. **Marena de la C. Hernández‐Lugo**: conceptualization, data curation, validation, writing – review and editing. **Carlos Ramos‐Galarza**: conceptualization, methodology, writing – review and editing.

## Funding

The authors have nothing to report.

## Conflicts of Interest

The authors declare no conflicts of interest.

## Ethics Statement

The study protocol received approval from the ethics committee of the Department of Psychology at Universidad Central ‘Marta Abreu’ de Las Villas. All procedures conducted in this study adhered to the ethical standards outlined in the 1964 Helsinki Declaration. Informed consent was obtained from all participants involved in the study.

## Data Availability

The dataset for this study is available at the Mendeley Repository: https://data.mendeley.com/datasets/wf8dgfd6y8/1

## Appendix 1 Global Brain Health Survey

### 1.1

To assess willingness to undergo brain health testing and attitudes toward lifestyle change, the study used selected items from the *Global Brain Health Survey* (GBHS; Budin‐Ljøsne et al. 2020).

Below, we describe each analyzed question, including its response format and selection rules.

**Q1. Willingness to take a brain health test**

Participants were asked:

“*Imagine a simple brain health test to learn about risk of developing a brain disease. Would you wish to take such a test?*”

Response options (single choice, 4‐point Likert scale):

- Yes — definitely
- Yes — probably
- No — probably not
- No — definitely not

**Q2. Willingness to test for unpreventable or untreatable diseases**

Participants were asked:

“*Would you take a test even if it provides information about a disease that cannot be prevented or treated?*”

Response options (single choice, 4‐point Likert scale):

- Yes — definitely
- Yes — probably
- No — probably not
- No — definitely not

**Q3. Reasons for taking a brain health test**

Participants who indicated willingness to take a brain health test were asked:

“*Why would you take a brain health test?*”

Response options (multiple choice; participants could select **more than one option**):

- •:To get information about my cognitive and mental health
- To determine my risk of developing a brain disease
- To respond if I am at risk
- To prepare myself for the future

**Q4. Reasons for NOT wanting to take a brain health test**

Participants who indicated unwillingness to take a test were asked:

“*Why would you NOT take a brain health test?*”

Response options (multiple choice; participants could select **more than one option**):

- I do not want to worry about something that may not happen.
- I do not want to know about a disease that could not be prevented or treated.
- I would be frightened by the result.
- There is nothing I can do for my brain health anyway.

**Q5. Likely reactions to brain health risk information**

Participants were asked:

*“Imagine you undergo a brain health test, and it shows that you have a risk of developing brain disease. What would be your most likely reaction?”*

Response options (each item rated separately; 4‐point Likert scale):

- I would seek professional help (e.g., my doctor).
- I would seek advice from family and friends.
- I would seek information online or at the library.
- I would change my lifestyle if required.
- I would plan for the future.

Response categories:

- Definitely yes
- Fairly likely
- Fairly unlikely
- Definitely not

For analysis, **“Definitely yes” and “Fairly likely” responses were combined**, consistent with prior GBHS studies.

**Q6. Willingness to change specific lifestyle behaviors**

Participants were asked:

*“Your doctor tells you that you can reduce your risk of developing a brain disease by changing your lifestyle. How likely are you to do any of the following?”*

Items included:

- Change eating habits
- Exercise more
- Sleep better
- Engage in more relaxing activities
- Participate in more brain‐stimulating activities
- Reduce alcohol consumption
- Reduce smoking
- Socialize more

Response options (each item rated separately; 4‐point Likert scale):

- Definitely yes
- Fairly likely
- Fairly unlikely
- Definitely not

Analyses focused on participants who reported not already engaging frequently in each behavior.

**Q7. Motivations for lifestyle change**

Participants were asked:

“*What would motivate you to change your lifestyle to improve brain health?*”

Response options (multiple choice; participants could select **more than one option**), including:

- Increasing frequency of brain problems (e.g., memory loss, difficulty concentrating)
- Diagnosis of a brain disease
- Diagnosis of a close friend or relative
- Advice from a doctor
- Support from family or friends
- Knowing lifestyle changes are beneficial
- Interventions being fun or easy to follow
- Interventions being affordable

**Q8. Barriers to lifestyle change**

Participants were asked:

“*What would prevent you from changing your lifestyle for your brain health?*”

Response options (multiple choice; participants could select **more than one option**):

- Lack of information
- Lack of motivation
- Lack of time
- Financial cost
- Having to give up enjoyable activities
- Uncertainty about effectiveness
- Having to do activities I do not enjoy
- Having to make changes alone

**Q9. Background brain‐health–related items**

Participants also answered background questions regarding:

- How often they think about their brain health
- Whether they have cared for a family member with a brain disease
- Previous participation in brain health research
- Presence of disability or illness affecting work

These items were used to characterize the sample and explore associations with willingness and reactions.

**Demographic variables**

The survey collected standard demographic information, including:

- Age (years; categorized as 18–25, 26–35, 36–45)
- Gender
- Educational level
- Employment status
- Healthcare experience
